# ^18^F-FDG uptake in the colon is modulated by metformin but not associated with core body temperature and energy expenditure

**DOI:** 10.1371/journal.pone.0176242

**Published:** 2017-05-02

**Authors:** Lonneke Bahler, Frits Holleman, Man-Wai Chan, Jan Booij, Joost B. Hoekstra, Hein J. Verberne

**Affiliations:** 1 Internal Medicine, Academic Medical Center, Amsterdam, The Netherlands; 2 Nuclear Medicine, Academic Medical Center, Amsterdam, The Netherlands; Rush University Medical Center, UNITED STATES

## Abstract

**Purpose:**

Physiological colonic ^18^F-fluorodeoxyglucose (^18^F-FDG) uptake is a frequent finding on ^18^F-FDG positron emission tomography computed tomography (PET-CT). Interestingly, metformin, a glucose lowering drug associated with moderate weight loss, is also associated with an increased colonic ^18^F-FDG uptake. Consequently, increased colonic glucose use might partly explain the weight losing effect of metformin when this results in an increased energy expenditure and/or core body temperature. Therefore, we aimed to determine whether metformin modifies the metabolic activity of the colon by increasing glucose uptake.

**Methods:**

In this open label, non-randomized, prospective mechanistic study, we included eight lean and eight overweight males. We measured colonic ^18^F-FDG uptake on PET-CT, energy expenditure and core body temperature before and after the use of metformin. The maximal colonic ^18^F-FDG uptake was measured in 5 separate segments (caecum, colon ascendens,—transversum,—descendens and sigmoid).

**Results:**

The maximal colonic ^18^F-FDG uptake increased significantly in all separate segments after the use of metformin. There was no significant difference in energy expenditure or core body temperature after the use of metformin. There was no correlation between maximal colonic ^18^F-FDG uptake and energy expenditure or core body temperature.

**Conclusion:**

Metformin significantly increases colonic ^18^F-FDG uptake, but this increased uptake is not associated with an increase in energy expenditure or core body temperature. Although the colon might be an important site of the glucose plasma lowering actions of metformin, this mechanism of action does not explain directly any associated weight loss.

## Introduction

Obesity and diabetes mellitus type 2 (DM2) are health problems with a tremendous impact and a still increasing prevalence. Many attempts have been made to combat obesity and DM2, however the current therapies are lacking in effectivity [1].

^18^F-fluorodeoxyglucose (^18^F-FDG) positron emission tomography computed tomography (PET-CT) pinpoints areas with high glucose turnover. In a retrospective analyses, approximately 50% of the patients that underwent a diagnostic ^18^F-FDG PET-CT showed high ^18^F-FDG uptake in the colon, suggesting that the colon has a comparatively high glucose consumption [2, 3]. Indeed, in pigs it has been shown that intestinal glucose uptake can be quantified, in vivo, by performing ^18^F-FDG PET-CT [4].

Metformin, a drug widely used in the treatment of DM2, is associated with moderate weight loss[5–7]. Interestingly, retrospective analyses have shown that patients using metformin tend to have a diffusely increased ^18^F-FDG uptake in the colon but this association has not yet been prospectively evaluated [8–11]. In addition, although this increase in colonic glucose uptake is associated with metformin use, the exact causal relation between colonic glucose uptake and metformin is unknown. However an increased glucose consumption by the colon might contribute to the positive effects of metformin on weight.

If the colon increases the glucose consumption under influence of metformin, energy expenditure is expected to increase. Putatively, an increased muscle peristalsis would increase glucose demand and thereby ^18^F-FDG uptake. This might cause an increase in core temperature (especially in the colon) as increased muscle activity results in the production of heat. Apart from metformin there are no other known modulators that increase ^18^F-FDG uptake in the colon.

Therefore, the aim of this study was to determine prospectively whether metformin increases ^18^F-FDG uptake in the colon. We further wanted to assess whether the ^18^F-FDG uptake was associated with an increase in energy expenditure and/or core body temperature.

## Materials and methods

The Medical Ethics Committee of the Academic Medical Center of the University of Amsterdam approved the study protocol (S1 File) on July the 22^nd^ 2015 and the study was conducted according to the Declaration of Helsinki. All subjects gave written informed consent after oral and written explanation of the procedures performed during the study. Participants were recruited by public advertisement. This study was registered on www.clinicaltrials.gov with the registration number NCT02783053. The registry of this study was completed after the first recruitment of subjects because of a delay in finalizing the protocol on the website. However, the authors confirm that all ongoing and related trials for this drug/intervention are registered.

The manuscript was written according to the trend statement checklist (S2 File).

### Participants

**Fig 1:**
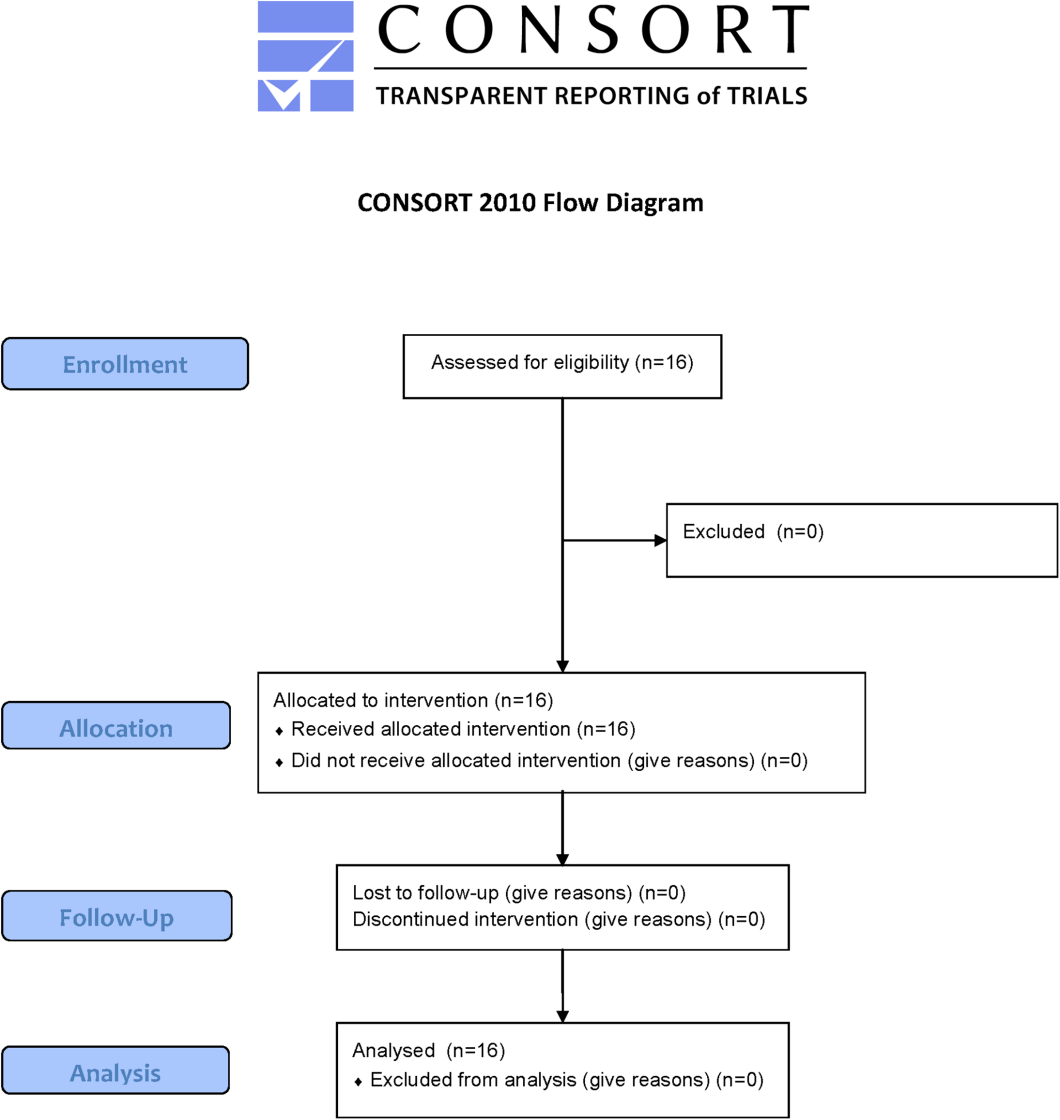
Consort flowchart. Flow chart of subjects completing each stage of the study. We screened and included 16 subjects (eight overweight (body mass index [BMI], > 28 kg/m^2^) and eight lean (BMI, <24 kg/m^2^). All 16 subjects completed the study and were included in the analysis.

For this study, we screened and included16 healthy, European decent male volunteers with an age >50 years: eight overweight (body mass index [BMI], > 28 kg/m^2^) and eight lean (BMI, <24 kg/m^2^). Volunteers were recruited and finalized the study protocol between October the 15^th^ 2015 and June the 9^th^ 2016. Healthy subjects were defined as subjects without any current or ongoing diseases (e.g. diabetes, infections, cancer etc.) In addition subjects were required not to use any medication or drugs, including anti-DM drugs. Baseline characteristics, including length, weight, waist circumference, medical history (including medication use), laboratory measurements (kidney function and liver function), were obtained during the baseline visit. All subjects completed all study visits and all subjects were included in the analysis (Fig 1).

### Sample size calculation

We based our sample size on a retrospective study[3], we aimed to find an increase in SUV_max_ of 2,8 g/L in the colon. We calculated that a sample size of 8 would have had 80% power to detect a difference in means of 2,8 (e.g. a First condition mean of 2,7 SUV_max_ and a Second condition mean of 5,5 SUV_max_), assuming a standard deviation of differences of 2,24, using a paired t-test with a 0,05 two-sided significance level.

### Study design

This was an open label, non-randomized, prospective mechanistic study investigating whether an increase in ^18^F-FDG uptake in the colon resulted in energy disposal by increasing energy expenditure and/or core temperature. Subjects were investigated on two study visits, one before and one after using metformin. Thousand mg of metformin orally was used for seven days, 500 mg in the morning and 500 mg in the evening. We based our dosing regimen on the fact that discontinuation of metformin for two days have been shown to significantly reduce colonic ^18^F-FDG uptake.[9].

When subjects were eligible for inclusion, the two study visits were planned. The study visits were separated by a two week interval. On the first visit, subjects received the metformin tablets from the PhD student along with the metformin instructions and clear oral instructions how to use the tablets and the potential side effects. On both study visits, subjects arrived after at least a six hour fast at the Clinical Trial Unit. The evening before the last visit, subjects used their last metformin tablet. Compliance was checked by pill count and anamnesis. After arrival, the equipment to measure the core body temperature was applied. Weight was measured with the subjects wearing only underwear and on the same calibrated mechanical scale (SECA) to the nearest 100 g. Height and waist circumference were recorded to the nearest 0.01 m.

Subsequently, subjects were rested on a bed and energy expenditure (EE) was measured for 20 minutes. After the EE measurement, subjects were again rested on a bed and 100 MBq of ^18^F-FDG was administered intravenously. One hour after the administration of ^18^F-FDG a PET-CT imaging of the abdomen was performed.

### Core body temperature measurements

Core body temperature was measured using a VitalSense® Core temperature capsule, and data were analysed using Equivital^TM^ Manager (Hidalgo Limited, Cambridge, United Kingdom) core temperature pill. The mean transit time of the colon was estimated at approximately 30 hours [12, 13]. Therefore, subjects had to ingest the activated core temperature pill twenty-four hours prior to the visits to assure that the pill was located in the colon at the time the measurements took place. Core temperature information was retrieved after arrival at the Clinical Trial Unit by the core temperature device which receives the signals from the core pill.

### Energy expenditure (EE)

Energy expenditure (kcal/day) was measured via indirect calorimetry, using a ventilated hood system (Vmax® encore, Becton, Dickinson and Company, United States) for approximately 20 minutes in a supine position. During these measurements, the respiratory quotient (RQ = CO_2_ during expiration / O_2_ usage) was also obtained as an indirect measure of calories used. Subjects were not allowed to move or talk during the measurements.

### ^18^F-FDG uptake in the colon

^18^F-FDG PET-CT scans were obtained using a Gemini time-of-flight multidetector helical PET-CT scanner (Philips Medical Systems, Eindhoven, the Netherlands). Subjects were rested on a bed in a warm room (25°C) an half hour after the administration of ^18^F-FDG in order to minimize the uptake of ^18^F-FDG in muscles and brown adipose tissue. PET images were acquired one hour after the administration of ^18^F-FDG and included diaphragm to the pelvis. For attenuation correction and anatomical colocation, a low dose CT imaging (120 kV, 40 mAs) was consecutively performed.

### PET CT image analysis

The ^18^F-FDG uptake in the colon on the PET-CT images was scored visually and by generating volumes of interest (VOI) using the software program Hybrid Viewer (Hermes Medical Solutions, Stockholm, Sweden). For the analysis, the colon was divided into 5 segments: the distal ileum (later referred to as ileum), the cecum and ascending colon (later referred to as cecum), the hepatic flexure and the transverse colon (later referred to as transverse colon), the splenic flexure and the descending colon (later referred to as colon descendens) and the sigmoid colon ending at the recto sigmoid junction (later referred to as sigmoid), according to our previously published method [3].

Visual assessment of the colonic uptake of ^18^F-FDG was performed according to the 4-point scale described by *Gontier et al*.[10], using the hepatic ^18^F-FDG uptake as a reference (i.e., 1 = lower, 2 = similar, 3 = moderately higher and 4 = intense and diffuse increased uptake).

In colonic segments with a visual grading of ≥ 3, volumes of interest (VOIs) were generated. After generating the VOI, the volume (mL), maximal and mean ^18^F-FDG uptake (standardized uptake values, SUV_max_ and SUV_mean_ respectively) were determined. In colonic segments with a visual grading of ≤ 2, the software program was not able to generate VOIs. In these segments, SUV_max_ was assessed using the option “quick-ROI”. The SUV_max_ is defined as ^18^F-FDG activity in becquerel per milliliter within the ROI divided by injected dose in becquerel per gram of body weight. As a reference the ^18^F-FDG uptake in the liver was determined (i.e. SUV_max_ in a VOI of 10 consecutive transverse liver ^18^F-FDG PET-CT slices), according to our previously published method [3]. The readers were blind as to whether metformin was used or not when analysing the images.

### Objectives and hypothesis

Our first aim was to confirm prospectively that metformin significantly increases ^18^F-FDG uptake in the colon. Our secondary aims were to assess whether the increased ^18^F-FDG uptake was correlated with an increase in energy expenditure and/or core body temperature.

We hypothesized that metformin could increase ^18^F-FDG uptake in the colon. Furthermore, we hypothesized that this increase in ^18^F-FDG uptake in the colon would result in an increased energy disposal in the form of an increase in energy expenditure and/or core body temperature.

Our primary outcome measure was therefore the increase in ^18^F-FDG uptake in the colon after the use of metformin to confirm the role of metformin in ^18^F-FDG uptake in the colon prospectively.

Our secondary outcomes were the correlation between the increase in ^18^F-FDG uptake and the increase in energy expenditure and/or core body temperature.

### Statistical analysis

Data are represented as median and interquartile range (IQR). A p-value <0.05 was considered as statistically significant. Differences in paired analysis were calculated using the Wilcoxon signed rank test. Differences between groups were calculated using the Mann Whitney U test.

The relative increase in colonic ^18^F-FDG uptake was calculated as (^18^F-FDG uptake post-exposure * 100%) / ^18^F-FDG uptake pre-exposure. For the statistical analysis, SPSS 20.0 was used. The database can be found as supporting file (S3 File).

## Results

In total, we included eight lean (22.1 [21.4–22.6] kg/m^2^, age 60 [54–66] years) and eight overweight males (BMI 31.3 [28.9–33.4] kg/m^2^, age 63 [53–68] years). There was no significant difference in age between the two groups. As expected, BMI, waist circumference and fasting plasma glucose were significantly higher in the overweight group (Table 1). All subjects tolerated the metformin well, there were no complaints of side effects.

**Table 1:** Baseline table.

	Lean	Overweight	p-value
**N**	8	8	
**Age (years)**	60 [54–66]	63 [53–68]	0.574
**Body Mass Index (kg/m**^**2**^**)**	22.1 [21.4–22.6]	31.3 [28.9–33.4]	<0.001
**Waist circumference (cm)**	88 [81–91]	111 [105–114]	<0.001
**Fasting plasma glucose (mmol/L)**	5.3 [5.0–5.6]	5.9 [5.6–6.9]	<0.001

Characteristics of subjects. Data presented as median [interquartile range]. BMI, body mass index. Differences between the groups were calculated with the Mann Whitney U test.

Due to technical failure of the device, core temperature measurements did not succeed in every patient. Core temperature measurements succeeded in seven out of eight lean subjects and two out of eight overweight subjects.

### Effect of metformin

**Fig 2:**
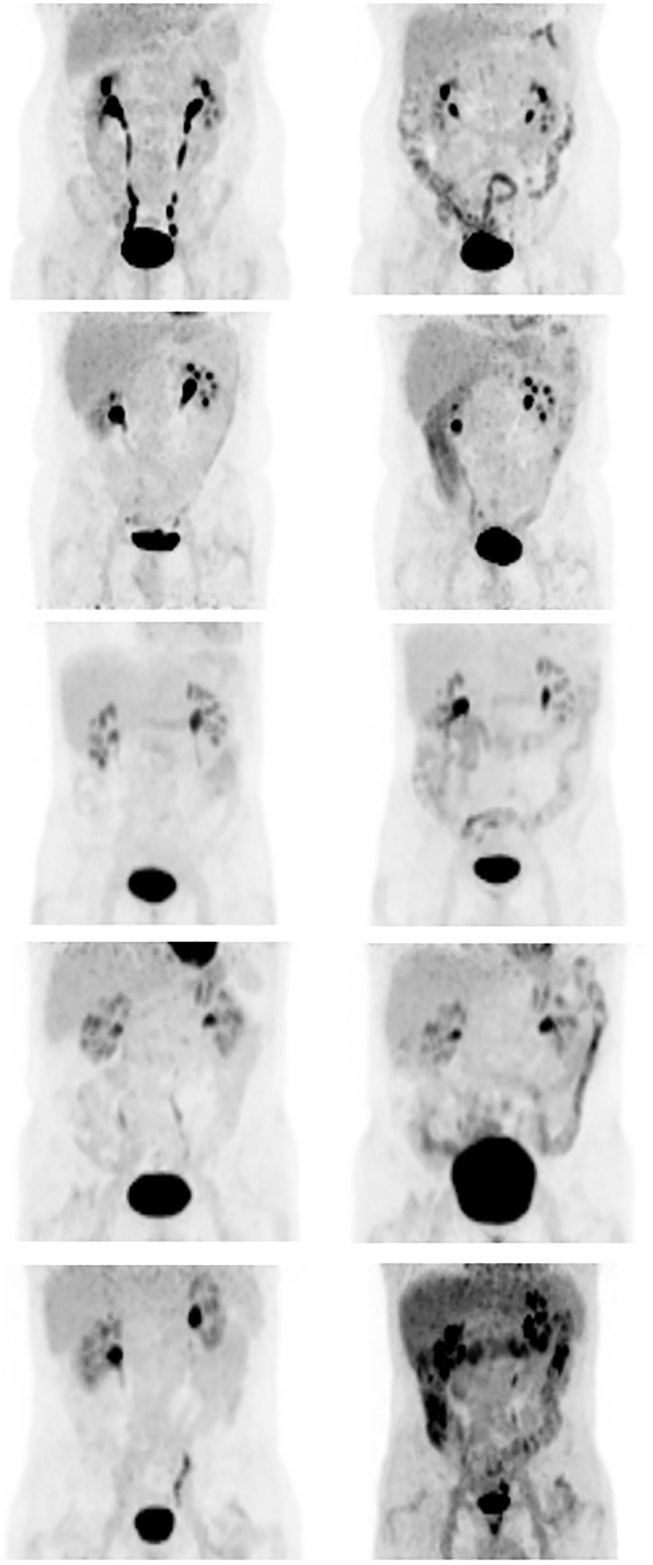
Typical cases. Five typical examples of ^18^F-FDG uptake in the colon before (left panel) and after (right panel) the administration of metformin. Please note the increased ^18^F-FDG uptake in the colon after metformin administration.

The ^18^F-FDG uptake increased significantly in all segments of the colon after metformin administration, both in the overall study population as well as when the subjects were stratified by BMI (Fig 2 and Table 2). There was no effect of metformin on mean core temperature, EE or RQ.

**Table 2:** Effect of metformin.

	All subjects			Lean			Overweight		
	Pre-exposure	Post-exposure	p-value	Pre-exposure	Post-exposure	p-value	Pre-exposure	Post-exposure	p-value
**Grade Total Colon**^**#**^	1.5 [1.0–2.0]	4.0 [3.0–4.0]	**0.001**	1.0 [1.0–2.0]	4.0 [3.25–4.0]	**0.016**	2.0 [1.0–2.0]	3.0 [2.25–4.0]	**0.014**
**Liver (SUV**_**max**_**)**	4.0 [3.5–4.1]	3.7 [2.7–4.6]	0.501	3.5 [2.7–3.7]	3.1 [2.5–3.7]	0.779	4.1 [4.0–5.6]	4.3 [3.8–5.2]	0.483
**Liver (SUV**_**mean**_**)**	2.3 [2.1–2.6]	2.3 [1.8–2.6]	0.313	2.1 [1.9–2.3]	2.1 [1.8–2.3]	0.944	2.5 [2.4–2.8]	2.5 [2.0–2.6]	0.123
**Caecum (SUV**_**max**_**)**	1.9 [1.6–2.4]	3.2 [2.6–5.5]	**<0.001**	1.8 [1.6–1.9]	3.2 [2.8–6.2]	**0.012**	2.1 [1.6–2.8]	2.8 [2.5–3.9]	**0.017**
**Ascendens (SUV**_**max**_**)**	2.2 [1.7–3.1]	4.0 [3.1–5.6]	**0.002**	1.7 [1.6–3.3]	4.3 [3.9–5.6]	**0.017**	2.5 [2.2–3.1]	3.4 [2.6–6.5]	**0.028**
**Transversum (SUV**_**max**_**)**	1.7 [1.5–2.1]	3.0 [1.9–3.9]	**0.004**	1.6 [1.5–2.2]	3.1 [1.9–3.9]	**0.036**	1.7 [1.6–1.9]	2.8 [1.6–4.1]	**0.050**
**Descendens (SUV**_**max**_**)**	2.3 [1.9–2.6]	5.4 [2.8–7.6]	**<0.001**	2.1 [1.9–2.6]	6.4 [3.5–10.8]	**0.017**	2.4 [1.7–2.6]	4.6 [2.5–7.0]	**0.025**
**Sigmoid (SUV**_**max**_**)**	1.7 [1.5–3.1]	6.1 [5.1–8.9]	**<0.001**	1.6 [1.5–2.2]	6.9 [5.6–9.6]	**0.012**	2.4 [1.3–3.9]	5.7 [4.0–8.8]	**0.012**
**EE (kcal/day)**	1860 [1667–2062]	1897 [1723–2059]	0.877	1743 [1597–1867]	1725 [1553–1880]	0.263	2020 [1791–2138]	2056 [1940–2228]	0.674
**RQ**	0.86 [0.84–0.88]	0.86 [0.82–0.88]	0.501	0.85 [0.83–0.91]	0.86 [0.85–0.87]	0.779	0.85 [0.84–0.91]	0.83 [0.79–0.93]	0.263
Mean temp (°C)*	36.7 [36.5–37.0]	36.8 [36.3–37.0]	0.514	36.7 [36.3–36.9]	36.6 [36.4–37.0]	0.236	37.0 [36.8–37.0]	36.8 [36.6–36.8]	0.180

Data are presented as median [interquartile range]. Differences between the visits were calculated using the Wilcoxon signed rank test. SUV_max_: maximal standard uptake value, defined as activity in Becquerel per milliliter within region of interest divided by injected dose in Becquerel per gram of body weight.

^#^ Grading of total colon was obtained using the visual assessment score.

* Core body temperature measurements succeeded in 7/8 lean subjects and 2/8 overweight subjects.

However, temperature measurements failed in one out of eight of the lean subjects and in six out of eight overweight subjects. In these subjects, the device which receives the temperature data failed to receive data from the core temperature pill.

At baseline the ^18^F-FDG uptake in any of the segments of the colon did not significantly differ between the lean and overweight subjects. In addition, ^18^F-FDG uptake in any of the segments of the colon did not significantly differ between lean and overweight subjects after metformin use. Also the increase, both relative and absolute, in ^18^F-FDG uptake in the colon did not significantly differ between the lean and obese subjects after metformin(data not shown).

There was, however, a significant difference in the baseline SUV_max_ values of the liver between the lean and overweight subjects (3.5 [2.7–3.7] vs 4.1 [4.0–5.6], p = 0.038).

**Fig 3:**
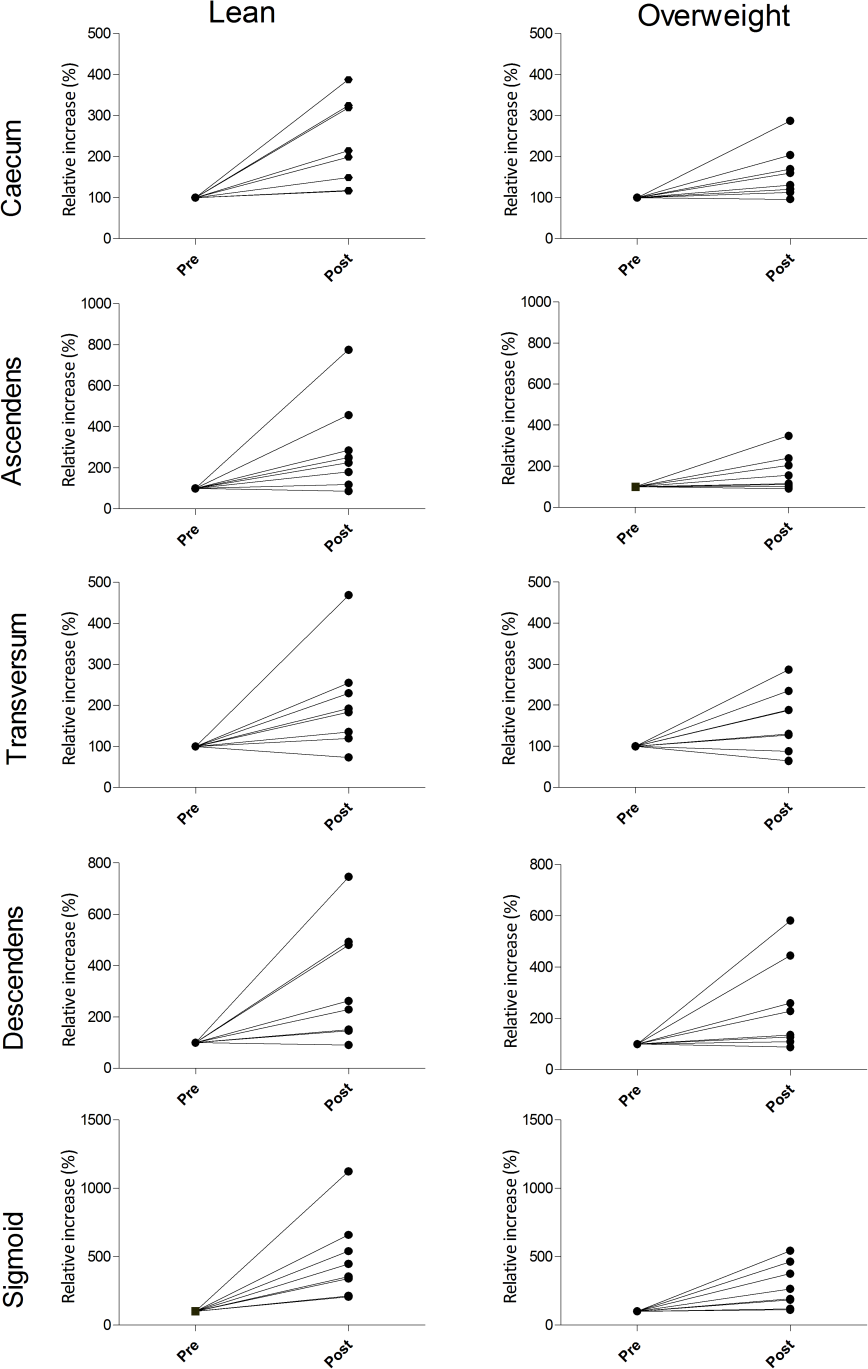
Relative increase of ^18^F-FDG uptake. The relative increase in 18F-FDG uptake in the separate segments of the colon in lean (left panel) and overweight (right panel) subjects. The relative increase was calculated as (^18^F-FDG uptake post-exposure * 100%) / ^18^F-FDG uptake pre-exposure.

In the sigmoid, the ^18^F-FDG uptake increased in all subjects after metformin administration. In the remaining segments, ^18^F-FDG uptake increased in almost every subject (Fig 3). There was no significant difference in absolute or relative increase in maximal ^18^F-FDG uptake between the lean or overweight subjects.

### Correlations between ^18^F-FDG uptake and BMI, EE and core body temperature

Pre-exposure, there were no significant correlations between ^18^F-FDG uptake in the colon (visual assessment of the colon, the sum of the visual assessment of 5 segments and maximal ^18^F-FDG uptake in the colon) and BMI, core body temperature (sub group analysis of subjects in which core body temperature measurements succeeded) or EE. Post-exposure, there was a significant inverse correlation between BMI and the visual assessment of the colon (ρ = -0.51; p = 0.04), and between EE and the visual assessment of the colon (ρ = -0.67; p <0.01), and EE and the maximal uptake of ^18^F-FDG in the colon (ρ = -0.53; p = 0.04). After correction for BMI, none of the correlations remained significant. (Table 3).

**Table 3:** Correlations between ^18^F-FDG uptake and parameters of energy disposal.

	**Pre- exposure**		**Post- exposure**	
	**Grade total colon**	**Colon SUV**_**max**_	**Grade total colon**	**Colon SUV**_**max**_
**BMI (kg/m**^**2**^**)**	ρ -0.01; p = 0.98	ρ 0.41; p = 0.12	**ρ -0.51; p = 0.04**	ρ -0.34; p = 0.20
Core Body Temperature (°C)*	ρ 0.10; p = 0.79	ρ 0.07; p = 0.87	ρ 0.29; p = 0.46	ρ 0.62; p = 0.08
**Energy Expenditure (kcal/day)**	ρ -0.20; p = 0.47	ρ 0.25; p = 0.36	**ρ -0.67; p <0.01**	**ρ -0.53; p = 0.04**
**After correction of BMI**	Pre- exposure		Post- exposure	
	Grade total colon	Colon SUV_max_	Grade total colon	Colon SUV_max_
Core Body Temperature (°C)*	ρ -0.16; p = 0.70	ρ -0.01; p = 0.98	ρ 0.36; p = 0.16	ρ 0.68; p = 0.06
**Energy Expenditure (kcal/day)**	ρ 0.02; p = 0.96	ρ -0.12; p = 0.77	ρ -0.46; p = 0.25	ρ -0.42; p = 0.30

Correlations between parameters. calculated with Spearman’s Rho. Grade total colon is calculated as the sum of the visual assessment of the separate segments of the colon according to the 4-point scale. Colonic SUV_max_ is the maximal uptake of ^18^F-FDG in the colon. SUV_max_: maximal standard uptake value. defined as activity in Becquerel per milliliter within region of interest divided by injected dose in Becquerel per gram of body weight.

* Core body temperature measurements n = 9/16 (7/8 lean subjects and 2/8 obese subjects).

### Correlations between the differences in ^18^F-FDG uptake and BMI, EE and core body temperature

**Fig 4:**
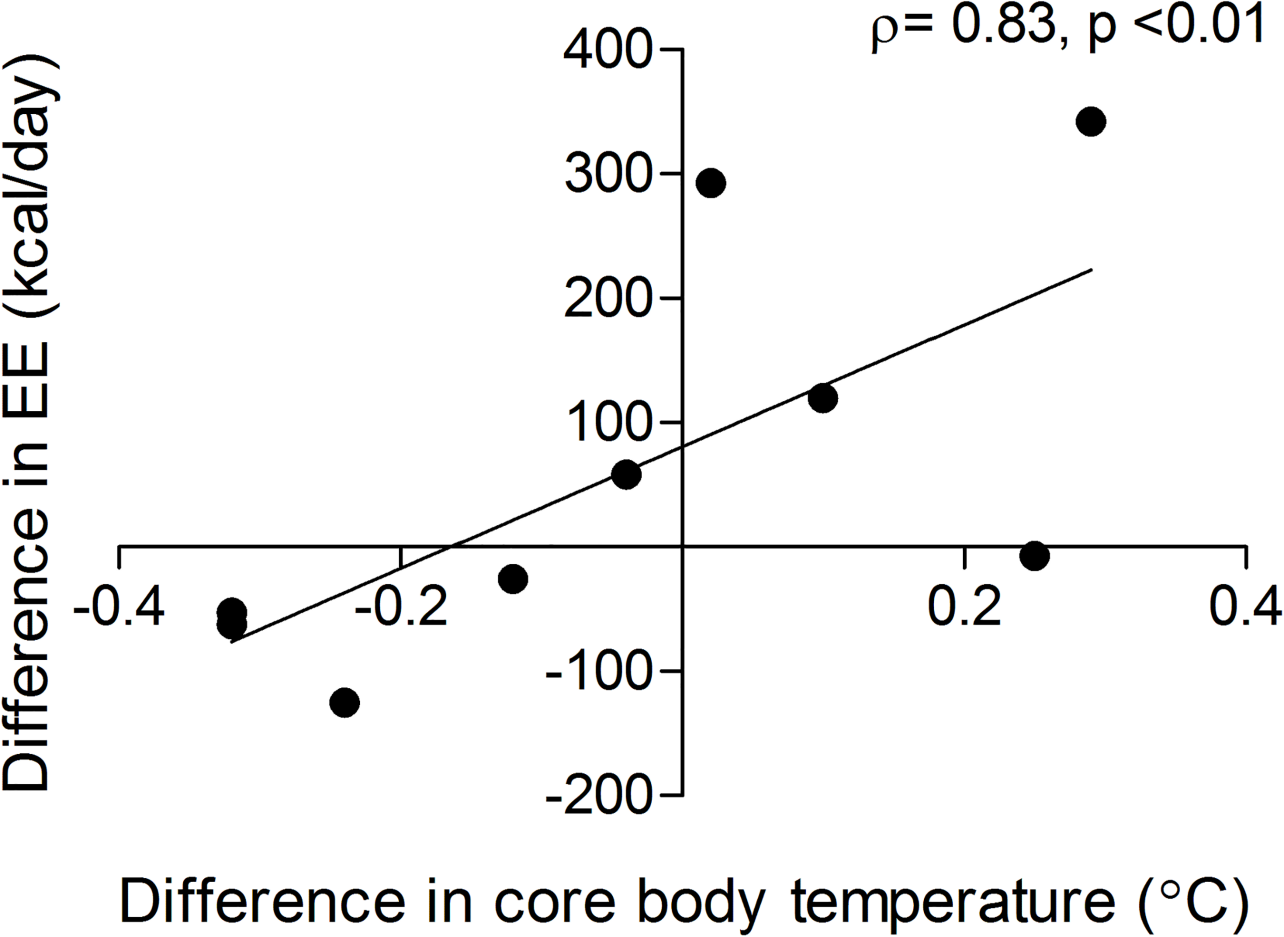
Correlations. Correlations between the difference in energy expenditure pre and post administration and the difference in core body temperature.

The difference in EE did not correlate significantly with any of the parameters of ^18^F-FDG uptake in the colon. The difference in core body temperature (sub group analysis of subjects in which core body temperature measurements succeeded) did not significantly correlate with any of the parameters of ^18^F-FDG uptake in the colon, but there was a significant correlation between the difference in EE and the difference in core temperature (Fig 4; ρ = 0.83; p = 0.006).

## Discussion

This is the first prospective study investigating the effect of metformin on ^18^F-FDG uptake in the colon and the association of ^18^F-FDG uptake in the colon with energy expenditure and/or core body temperature. We prospectively confirmed that the use of metformin significantly increases ^18^F-FDG uptake in the colon. However, this increase was not associated with an increase in EE or core body temperature.

Both in lean and overweight subjects without DM2, the administration of metformin resulted in a significant increase in colonic ^18^F-FDG uptake. This confirms the findings in retrospective observational studies which show an association between the use of metformin in patients with DM2 and ^18^F-FDG uptake [3, 8, 10]. Conversely, discontinuing the use of metformin in patients with DM2 has been shown to reduce the uptake of ^18^F-FDG in the colon significantly [9, 11].

It has been shown, in healthy pigs, that intestinal glucose uptake can be quantified, in vivo, by performing ^18^F-FDG PET-CT [4]. So, the increased ^18^F-FDG uptake after metformin administration reflects an increased glucose uptake in the colon. Indeed, the colon has been shown to be a site of increased glucose utilization during metformin treatment in mice and thereby contribute to the glucose lowering effect [14–17]. Whether this is due to upregulation of glucose transporters is still not entirely clear [18, 19].

The effect of metformin might be different in the intestine than in other tissues since metformin concentrations in the mucosa of the intestine exceed the concentrations in plasma and other tissues. After ingestion, the absorption of metformin from the small intestine is only partial and the concentration of metformin in the lumen remains high [20], approximately 30% of the metformin is excreted via faeces [21]. Furthermore, the uptake of metformin from the luminal surface of the enterocytes is relatively unhampered but efflux across the basolateral side is limited, resulting in accumulation of metformin in the epithelium [22]. Thus, the colon might have a significant role in the glucose lowering actions of metformin. In this respect, it is important to note that ^18^F-FDG is administered intravenously. Therefore ^18^F-FDG uptake in the colon reflects glucose uptake from the blood. Furthermore, glucose uptake from the lumen of the colon is found to be insignificant as compared to the uptake of glucose from the blood and therefore the uptake of glucose is considered close to zero [23]. Still, the underlying molecular mechanism whereby metformin increases the glucose uptake in the colon remains unclear [24].

Though the colonic ^18^F-FDG uptake increased significantly, there was no significant change in EE or core body temperature after metformin treatment. Important to note is the technical failure of the device which receives the temperature data, the device failed to receive data from the core temperature pill in one out of eight lean subjects and six out of eight obese subjects. Since the device failed mostly in obese subjects, it is conceivable that BMI hampered the signal transduction of the core temperature pill to the device. Nonetheless, the temperature data has to be interpreted carefully. However, there was no change of core temperature in the lean subjects before or after metformin treatment. So, the possibility that metformin changes core temperature is fairly small.

The lack of change in EE after metformin treatment is conform the results of an earlier study.[25] However, this group investigated was relatively small (n = 10) and there is a large variability in the effectiveness and the pharmacokinetics of metformin between patients [26]. Therefore, metformin might not influence EE and/or core body temperature on group level but there might be an association between these parameters and the metabolic activity of the colon. Especially since we found a significant correlation between the difference in EE (EE after metformin treatment–EE before metformin treatment) and the difference in core temperature (core temperature after metformin treatment–core temperature before metformin treatment). Indeed, EE and several parameters of metabolic activity of the colon (visual grading, volume and the maximal uptake of ^18^F-FDG) after the use of metformin, were inversely correlated. However, when considering the role of BMI in EE, these correlations might have been driven mainly by BMI. This assumption was confirmed by the fact that after correction for BMI none of the correlations remained significant.

Interestingly, we found a strong correlation between the difference in EE and core body temperature whereby in approximately half of the subjects, metformin caused a decrease in EE and core body temperature whereas in the other half, metformin caused an increase in EE and core body temperature. This might again reflect the differences in effectiveness of metformin between individuals [26].

Apart from these factors, there are many other factors might influence the ^18^F-FDG uptake in the colon. The colon is characterized by a complexity of systems amongst others, the gut microbiome. Recent reports show that metformin influences the composition of the microbiome,[27–29] which might indirectly account for changes in ^18^F-FDG uptake. Especially the role of butyrate producing bacteria and/or short chain fatty acids might be interesting to evaluate. However, while the gut microbiome is different between lean and obese subjects with type 2 diabetes mellitus[30] we did not find any significant differences in SUV values between the lean and obese subjects. Furthermore, as mentioned above, ^18^F-FDG is administered intravenously. Therefore ^18^F-FDG uptake in the colon reflects glucose uptake from the blood whereas the gut microbiome is likely to mainly influence the luminal side of the colon. Nonetheless, metformin modifies both the ^18^F-FDG uptake in the colon and influences the gut microbiome composition. If and how these system influence each other would be an interesting topic for future research.

We previously showed that the liver was a reliable reference for the visual assessment of the colonic uptake of ^18^F-FDG since the uptake of ^18^F-FDG did not increase with increased colonic ^18^F-FDG uptake.[3] However, in the current analysis we showed that the baseline SUV_max_ values of the liver were significantly higher in overweight subjects than lean subjects. Thus, the colonic ^18^F-FDG uptake in overweight subjects might be underestimated as compared to the lean subjects in the visual assessment. Although speculative, this difference in^18^F-FDG liver uptake between lean and overweight may be seen as an early marker of the metabolic syndrome in the overweight subjects.

Though the sample size of the groups was rather small, we found a very convincing increase in ^18^F-FDG uptake in the colon after the administration metformin both in lean and the overweight subjects. All segments showed a significant increase in ^18^F-FDG after the use of metformin. By performing multiple tests, we might have introduced a type I error. However, as our data are in line with the results of previous studies type I errors seem unlikely.[3, 9, 10].

Even though the ^18^F-FDG uptake increased significantly after metformin, we did not find a correlation with energy expenditure and/or core body temperature. The effect of metformin on EE and/or core body temperature might have been subtle considering the relatively small mass of smooth muscle in the colon. Also, energy expenditure measurements do not take into account anaerobic glucose utilization. So, to further clarify the effect, in vitro studies have to be performed to assess the effect of metformin on anaerobic glucose utilization and its association with ^18^F-FDG uptake.

Furthermore, the effect of metformin might have been too diverse (considering the intra individual differences in effectiveness of metformin) and therefore a larger sample size might have revealed an association between ^18^F-FDG uptake and energy expenditure and/or core body temperature.

Our subjects used metformin for two weeks. This might have been too short to induce significant changes. It is thought that the actions of metformin are attributed to phenotypic modifications in gut cells, occurring after a relatively long time rather than to the presence of therapeutic drug concentrations.[31] Nonetheless, we found a very convincing increase in colonic ^18^F-FDG uptake, so if there was an association with EE or core body temperature, we would have found this.

Another important limitation of this study is the failure of the core body temperature measurements in one out of eight lean subjects and in six out of eight overweight subjects, and therefore we cannot draw firm conclusions from our data and our findings are in need of replication. Interestingly, most of the core body temperature measurements failed in obese subjects. This might imply that the signal of the core body temperature pill was disturbed due to an increased BMI. If this was the case, in future, a core body temperature pill might not be the best method to measure core body temperature in obese subjects. However, up until now, the core body temperature pill is still the most reliable method to measure the colonic temperature.[32] Rectal temperature would underestimate the colonic temperature.[33].

## Conclusions

Metformin significantly increases ^18^F-FDG uptake in the colon and this might potentially play an important role in the glucose lowering actions of metformin. However, the increase in ^18^F-FDG uptake in the colon is not associated with either a significant increase in EE or in core body temperature.

## Supporting information

S1 File Research protocol. Supporting file containing the research protocol.
(PDF)

S2 File Trend statement checklist. Supporting file containing the trend statement checklist.
(PDF)

S3 File Database. Supporting file containing the database.
(SAV)
