# Supplementary material for: 18F-FDG uptake in the colon is modulated by metformin but not associated with core body temperature and energy expenditure
Source: PLoS One. 2017 May 2;12(5):e0176242. doi: 10.1371/journal.pone.0176242 (PMC5413044; doi:10.1371/journal.pone.0176242)
Supplement: S1 File — Supporting file containing the research protocol. (PDF) [file pone.0176242.s001.pdf]

Metformin and Core Temperature in Obese and lean Males (McTOM)

(July 2015)

Version 3.0

Investigators:

L. Bahler, J.B.L. Hoekstra, J. Booij, H.J. Verberne, F. Holleman

PROTOCOL TITLE *Metformin and Core Temperature in Obese Males (McTOM)*

**Protocol ID**	**Metformin and Core Temperature in Obese Males (McTOM)**
**Short title**	**McTOM**
**EudraCT number**	***N/A***
**Version**	**3.0**
**Date**	**July 2015**
**Coordinating investigator/project leader**	**F. Holleman, f.holleman@amc.uva.nl, 81 59173**
**Principal investigator(s) (in Dutch: hoofdonderzoeker/ uitvoerder)**	**F. Holleman, f.holleman@amc.uva.nl, 81 59173**
**Sponsor (in Dutch: verrichter/opdrachtgever)**	**Non-commercial, Stichting Asklepios; J.B.L. Hoekstra**
**Subsidising party**	
**Independent expert (s)**	**Dr. P.H.L.T. Bisschop**
**Laboratory sites <*if applicable*>**	***LACK***
**Pharmacy <*if applicable*>**	***KC***

PROTOCOL SIGNATURE SHEET

Name	Signature	Date
**Sponsor or legal representative:**<br>***<please include name and function>***<br><br>***<For non-commercial research,>***<br>**Head of Department:**<br>***Prof. Dr. J.A. Romijn***		
**[Coordinating Investigator/Project leader/Principal Investigator]:**<br>***Dr. F. Holleman, internist***		

TABLE OF CONTENTS

1. INTRODUCION and RATIONALE 9
2. OBJECTIVES 10
3. STUDY DESIGN 11
4. STUDY POPULATION 13
   4.1 Population (base) 13
   4.2 Inclusion criteria 13
   4.3 Exclusion criteria 13
   4.4 Sample size calculation 13
5. TREATMENT OF SUBJECTS 14
   5.1 Investigational product/treatment 14
   5.2 Use of co-intervention (if applicable) 14
   5.3 Escape medication (if applicable) 14
   N/A 14
6. NON-INVESTIGATIONAL PRODUCT 15
   6.1 Name and description of non-investigational product(s) 15
   6.2 Summary of findings from non-clinical studies 15
   6.3 Summary of findings from clinical studies 15
   6.4 Summary of known and potential risks and benefits 15
   6.5 Description and justification of route of administration and dosage 15
   6.6 Dosages, dosage modifications and method of administration 15
   6.7 Preparation and labelling of Investigational Medicinal Product 15
   6.8 Drug accountability 15
7. METHODS 16
   7.1 Study parameters/endpoints 16
      7.1.1 Main study parameter/endpoint 16
      7.1.2 Secondary study parameters/endpoints (if applicable) 16
      7.1.3 Other study parameters (if applicable) 16
   7.2 Study procedures 16
   7.3 Withdrawal of individual subjects 16
      7.3.1 Specific criteria for withdrawal (if applicable) 17
   7.4 Replacement of individual subjects after withdrawal 17
   7.5 Follow-up of subjects withdrawn from treatment 17
   7.6 Premature termination of the study 17
8. SAFETY REPORTING 18
   8.1 Section 10 WMO event 18
   8.2 AEs, SAEs and SUSARs 18
      8.2.1 Adverse events (AEs) 18
      8.2.2 Serious adverse events (SAEs) 18
      8.2.3 Suspected unexpected serious adverse reactions (SUSARs) 19
   8.3 Annual safety report 20
   8.4 Follow-up of adverse events 20

9. STATISTICAL ANALYSIS 21
9.1 Primary study parameter(s) 21
9.2 Secondary study parameter(s) 21
9.3 Other study parameters 21
9.4 Interim analysis (if applicable) 21
10. ETHICAL CONSIDERATIONS 22
10.1 Regulation statement 22
10.2 Recruitment and consent 22
10.3 Objection by minors or incapacitated subjects (if applicable) 22
10.4 Benefits and risks assessment, group relatedness 22
10.5 Compensation for injury 22
10.6 Incentives (if applicable) 23
11. ADMINISTRATIVE ASPECTS, MONITORING AND PUBLICATION 24
11.1 Handling and storage of data and documents 24
11.2 Amendments 24
11.3 Annual progress report 24
11.4 End of study report 24
11.5 Public disclosure and publication policy 25
12. STRUCTURED RISK ANALYSIS 26
12.1 Potential issues of concern 26
12.2 Synthesis 27
13. REFERENCES 27

LIST OF ABBREVIATIONS AND RELEVANT DEFINITIONS

**ABR**	**ABR form, General Assessment and Registration form, is the application form that is required for submission to the accredited Ethics Committee (In Dutch, ABR = Algemene Beoordeling en Registratie)**
**AE**	**Adverse Event**
**AR**	**Adverse Reaction**
**CA**	**Competent Authority**
**CCMO**	**Central Committee on Research Involving Human Subjects; in Dutch: Centrale Commissie Mensgebonden Onderzoek**
**CV**	**Curriculum Vitae**
**DM2**	**diabetes mellitus type 2**
**EE**	**energy expenditure**
**^{18}F-FDG**	**^{18}F-fluorodeoxy-d-glucose**
**GCP**	**Good Clinical Practice**
**IB**	**Investigator’s Brochure**
**IC**	**Informed Consent**
**IMP**	**Investigational Medicinal Product**
**IMPD**	**Investigational Medicinal Product Dossier**
**METC**	**Medical research ethics committee (MREC); in Dutch: medisch ethische toetsing commissie (METC)**
**PET CT**	**positron emission tomography computed tomography**
**(S)AE**	**(Serious) Adverse Event**
**SPC**	**Summary of Product Characteristics (in Dutch: officiële productinfomatie IB1-tekst)**
**Sponsor**	**The sponsor is the party that commissions the organisation or performance of the research, for example a pharmaceutical company, academic hospital, scientific organisation or investigator. A party that provides funding for a study but does not commission it is not regarded as the sponsor, but referred to as a subsidising party.**
**SUSAR**	**Suspected Unexpected Serious Adverse Reaction**
**Wbp**	**Personal Data Protection Act (in Dutch: Wet Bescherming Persoonsgevens)**
**WMO**	**Medical Research Involving Human Subjects Act (in Dutch: Wet Medisch-wetenschappelijk Onderzoek met Mensen**

SUMMARY

Rationale: Obesity and diabetes mellitus type 2 (DM2) are health problems with a tremendous impact. Many attempts have been made to combat obesity and DM2, however, a breakthrough therapy is still lacking.
Obesity is the result of an imbalance between energy intake and energy expenditure. ^{18}F-fluorodeoxy-d-glucose (^{18}F-FDG) positron emission tomography computed tomography (PET-CT) pinpoint areas with high glucose turnover. Physiological ^{18}F-FDG accumulation is frequently observed in the colon. Therefore, the colon might play an important role in increasing energy expenditure by consuming calories. However, the possibility of the colon as an energy dissipating tissue has not yet been explored. The colon could become an interesting new target of research to find a method to combat obesity.
Metformin is one of the few drugs in the treatment of DM2 that is associated with moderate weight loss. Interestingly, patients using metformin show an increased ^{18}F-FDG-uptake in the colon. Whether this higher uptake of glucose also cause an increase in core temperature and/or an increase in energy expenditure is not known. The cause for this increase in glucose uptake in the colon by metformin use is unknown. Also, it is unknown whether this increase in glucose uptake results in an increased energy expenditure and/or an increase in core body temperature.
Objective: In this study we aim to investigate the influence of metformin (500 mg 1/day) on temperature in the colon, glucose uptake in the colon and energy expenditure in healthy lean (BMI < 24kg/m2) or obese subjects (BMI>28kg/m2). We will measure ^{18}F-FDG uptake in the colon, temperature in the colon, insulin sensitivity and energy expenditure before after using metformin.
Primairy objective: To investigate whether metformin increases ^{18}F-FDG uptake in healthy, lean and obese males.
Secondary Objectives:

1) To investigate whether metformin increases core body temperature in healthy, lean and obese males and whether this correlates with respectively ^{18}F-FDG uptake.
2) To investigate whether metformin increases energy expenditure in healthy, obese males and whether this correlates with respectively ^{18}F-FDG uptake.
3) To investigate whether metformin increases core temperature in healthy, obese males and whether this correlates with respectively ^{18}F-FDG uptake and energy expenditure.
4) To investigate whether metformin increases lactate in healthy obese males and whether there is a correlation between lactate and respectively ^{18}F-FDG uptake, core body temperature and energy expenditure.

Study design: Non-randomized interventional study

Study population: 16 healthy male volunteers (aged > 50 years, 8 obese BMI> 28 kg/m2 and 8 lean BMI<24 kg/m2) will be recruited. The volunteers will be recruited by advertisement in local paper and internet (via social media), covering the direct surroundings of our clinic (Amsterdam) and by advertisement at the medical faculty of the AMC.

Intervention (if applicable): Participants will use metformin during 7 days

Main study parameters/endpoints: Difference in ^{18}F-FDG uptake in the colon before and after using metformin.

Nature and extent of the burden and risks associated with participation, benefit and group relatedness: Included subjects will visit the AMC hospital on 3 occasions.

Visit 1: Informed consent, medical history, vital signs, laboratory measurements. Total blood drawn: 60 ml. Energy expenditure measurement after 60 minutes rest. Core body temperature measurement (core body pill, tympanic measurement and skin measurement). ^{18}F-FDG PET CT. Providing the metformin, start using metformin.

Visit 2 (wo weeks after visit 1): Energy expenditure after 60 minutes rest. Core body temperature measurement(core body pill, tympanic measurement and skin measurement). ^{18}F-FDG PET CT.

There is no direct benefit for the volunteers. The resulting dose from the radioactive tracers + the scans is 7.8 mSv. The placement of an intravenous canula can be an unpleasant experience and there is a small chance of developing flebitis at the site of the intravenous canula. While using metformin, the subjects can encounter side effects of the metformin. This research will provide insight into the working mechanism of metformin and its potential role in the treatment of obesity.

1. INTRODUCION and RATIONALE

Obesity and diabetes mellitus type 2 (DM2) are health problems with a tremendous impact. Many attempts have been made to combat obesity and DM2, however, a breakthrough therapy is still lacking[1].

Obesity is the result of an imbalance between energy intake and energy expenditure. ^{18}F-fluorodeoxy-d-glucose (^{18}F-FDG) positron emission tomography computed tomography (PET-CT) pinpoint areas with high glucose turnover. Physiological ^{18}F-FDG accumulation is frequently observed in the colon,[2] however, the possibility of the colon as an energy dissipating tissue has not yet been explored. Considering the high uptake of ^{18}F-FDG in the colon, the colon could have a positive effect on energy balance. Therefore, the colon could become an interesting new target of research to find a method to combat obesity.

Metformin is one of the few drugs in the treatment of DM 2 that is associated with moderate weight loss.[3-5]

Interestingly, patients using metformin are known to have increased ^{18}F-FDG-uptake in the colon.[6-9] A substantial number of subjects (e.g 42%) show an increased ^{18}F-FDG uptake without the use of metformin.[10] Nonetheless, in a matched case-control analysis, the use of metformin significantly increased physiological accumulation of ^{18}F-FDG in the colon.[6] Also, discontinuation of metformin in the 2 days before the ^{18}F-FDG PET-CT, significantly diminished the high ^{18}F-FDG uptake in the colon.[7] Indeed, the colon has been shown to be a site of increased glucose utilization during metformin treatment in mice.[11,12] The cause for this increase in glucose uptake in the colon by metformin use is unknown. A possible explanation for increased glucose uptake might be the shift from aerobic to anaerobic glucose metabolism due to the inhibition of the mitchondrial respiratory complex I during metformin use.[13] This mechanism has been proposed as a possible cause of changed intestinal glucose handling and increased production of lactate. This less efficient metabolism might in turn lead to increased heat dissipation. It is unknown whether this increase in glucose uptake in the colon results in an increased energy expenditure and/or an increase in core body temperature.

When core body temperature is measured using a temperature pill, with stable surrounding conditions, core body temperature changes only with very small steps (e.g. a range of 0,2 °C). Furthermore, the ingestible temperature pill has been shown to be a valid manner to measure core temperature.[14] However, in this study we will investigate whether metformin changes the core temperature significantly.

Therefore, we want to measure core temperature, energy expenditure and lactate in lean and obese healthy subjects before and after using metformin.

Furthermore, metformin is known to decrease variables of insulin resistance such as fasting insulin and HOMA-indexes, also among obese adolescents with no associated disease.[15] In

adults with impaired glucose tolerance it has been shown that lifestyle changes can prevent DM2[16]. However, metformin has also been shown to prevent DM2 in adults with impaired glucose tolerance.[5] Therefore, we want to measure insulin sensitivity in obese healthy subjects before and after using metformin using a homa index.

Aim of the study

The aim of this study is to determine whether metformin modifies the metabolic activity of the colon by an increased glucose uptake and thereby increases temperature. Furthermore, we want to measure energy expenditure, lactate concentration and core body temperature before and after metformin use.

2. OBJECTIVES

Primairy objective: To investigate whether metformin increases ^{18}F-FDG uptake in healthy, lean and obese males.

Secondary Objectives:

1) To investigate whether metformin increases core body temperature in healthy, lean and obese males and whether this correlates with respectively ^{18}F-FDG uptake.
2) To investigate whether metformin increases energy expenditure in healthy, obese males and whether this correlates with respectively ^{18}F-FDG uptake.
3) To investigate whether metformin increases core temperature in healthy, obese males and whether this correlates with respectively ^{18}F-FDG uptake and energy expenditure.
4) To investigate whether metformin increases lactate in healthy obese males and whether there is a correlation between lactate and respectively ^{18}F-FDG uptake, core body temperature and energy expenditure.

3. STUDY DESIGN

In this non-randomised intervention study we will study the influence of metformin (1000 mg/day (500 mg in the morning, 500 mg in the evening) during 1 week) on 18F-FDG uptake in the colon. The main outcome will be difference in 18F-FDG uptake. Furthermore, we will measure core body temperature, energy expenditure and HOMA-index. 16 healthy volunteers of Caucasian origin will be included (8 lean, BMI < 24 kg/m2 and 8 obese BMI > 28 kg/m2) in this study. Subjects (n=16) will be investigated before and after using metformin (1000 mg/day during 1 week). Included subjects will visit the AMC hospital on three occasions. Below is a description of each visit.

Study visits and procedures

During the **first** study visit, participants will come to the AMC for screening to determine eligibility for the study. During this visit, informed consent will be obtained after oral and written explanation of the study. Subsequently, length, weight, waist circumference and medical history will be acquired. In total, a maximum of 20 ml of blood will be drawn to perform laboratory measurements needed to determine eligibility for the study(kidney function and liver function). The chronic use of drugs and/or medication will be asked at the participant. If eligible, the second visit will be scheduled.

The **second and third visit** are similar. Core body temperature will be measured using a body core pill and tympanic temperature will be measured every 15 minutes. Blood will be drawn to measure insulin sensitivity, in total 40 ml of blood will be drawn to be able to perform blood tests and as stored serum. After 60 minutes an energy expenditure (EE) will be performed using indirect calorimetry. Glucose uptake in the colon will be evaluated using ^{18}F-FDG PET CT.

After the **second** visit, subjects will receive the metformin and information on how to use this.

Flow chart*; overview of the procedures that subjects will undergo in the course of the study.*

Visit 1. (screening)
Informed consent, evaluation of eligibility, medical history, laboratory measurements.

↓

Visit 2.
Weight, length, waist circumference, blood drawing, core body temperature, EE, ^{18}F-FDG PET CT. Start using metformin

↓

Visit 3.
Weight, length, waist circumference, blood drawing, core body temperature, EE, ^{18}F-FDG PET CT.

↓

End of study

4. STUDY POPULATION

STUDY POPULATION

4.1 Population (base)

16 healthy male volunteers (aged > 50 years, 8 lean (BMI<24 kg/m2) and 8 obese (BMI> 28 kg/m2)), will be recruited. Patients will be recruited by public call and advertisement in local papers, covering the wider surroundings of our clinic (Amsterdam).

4.2 Inclusion criteria

- Male
- Caucasian origin
- Subjects should be able and willing to give informed consent
- > 50 years old
- BMI< 24 kg/m2 or > 28 kg/m2

4.3 Exclusion criteria

- Renal failure (GFR< 60ml/min)
- Liver insufficiency (AST or ALT 3 times upper value)
- Chronic use of drugs or medication
- Diabetes mellitus
- Lactate acidosis or precoma diabeticum in medical history
- Acute or chronic diseases such as: dehydration, severe infection, shock, heartfailure, pulmonary insufficiency, recent heart attack
- Alcoholism

4.4 Sample size calculation

Based on a retrospective study we have recently performed,[L. Bahler, EASD 2014] we aim to find an increase in SUV of 2,8 g/L in the colon. This is an increase from grade 1 to grade 3 based on a visual scale designed by Gontier et al.[8] A sample size of 8 will have 80% power to detect a difference in means of 2,8 (e.g. a First condition mean of 2,7 SUV and a Second condition mean of 5,5 SUV), assuming a standard deviation of differences of 2,24, using a paired t-test with a 0,05 two-sided significance level. To allow for unexpected findings and to increase power we will include 11 subjects per arm.

5. TREATMENT OF SUBJECTS

5.1 Investigational product/treatment

Participants should use metformin twice a day (500mg in the morning and 500mg in the evening) during 7 days.

5.2 Use of co-intervention (if applicable)

During the entire study, participants should not take any kind of medication and/or drugs. If, in any case medication is used, they should informing the study-doctor.

5.3 Escape medication (if applicable)

N/A

6. NON-INVESTIGATIONAL PRODUCT

Metformin

6.1 Name and description of non-investigational product(s)

Metformin

6.2 Summary of findings from non-clinical studies

See Summary of Product Characteristics (SPC)

6.3 Summary of findings from clinical studies

See SPC chapter 5.1, page 7

6.4 Summary of known and potential risks and benefits

See SPC page 2, 6 & 9

6.5 Description and justification of route of administration and dosage

Normally, when metformin is given to a patient, the mean starting dose is 500mg 2 or 3 times a day. Therefore, we chose the lowest starting dose of 500 mg twice a day. Furthermore, metformin can only be given orally.

6.6 Dosages, dosage modifications and method of administration

Subjects will use the metformin 500mg twice a day, orally.

6.7 Preparation and labelling of Investigational Medicinal Product

Preparation and labelling of the investigational medicinal products will be done according to the relevant GMP guidelines (annex 13of the guideline Good Manufacturing Practice (2003/94/EG, via http://ec.europa.eu/health/files/eudralex/vol-4/2009_06_annex13.pdf))

6.8 Drug accountability

The “kenniscentrum geneesmiddelen onderzoek” at the AMC will order and distribute the metformin. The empty metformin packets will be returned to the “kenniscentrum geneesmiddelen onderzoek” after the local researcher has performed drug accountability.

7. METHODS

7.1 Study parameters/endpoints

7.1.1 Main study parameter/endpoint

Difference in 18F-FDG uptake as registered by 18F-FDG PET-CT before and after 7 days of daily metformin use.

7.1.2 Secondary study parameters/endpoints (if applicable)

- Difference in core body temperature before and after metformin use and the correlation with ^{18}F-FDG uptake in the colon.
- Difference in energy expenditure before and after metformin use and the correlation with ^{18}F-FDG uptake in the colon.
- Correlation between difference in core temperature and respectively ^{18}F-FDG uptake in the colon and energy expenditure, before, after 7 days of daily metformin use.
- Difference in lactate, before and after the use of metformin and the correlation between lactate and respectively ^{18}F-FDG uptake in the colon, core body temperature and energy expenditure.

7.1.3 Other study parameters (if applicable)

7.2 Study procedures

All included subjects will be asked about their medical history and undergo a brief physical examination (vital signs) at the screening visit. A detailed description of the study procedures has been given in the ‘Study Design’ section. Below you will find an overview of the invasive study procedures.

Invasive procedures:

- Blood sampling for laboratory test: liver function, kidney function
- 2 Placements of an intravenous canula at *visit 1, 2* blood sampling for laboratory test: plasma glucose, insulin and lactate
- Taking the body core temperature pill
- ^{18}F-FDG PET-CT

Total amount of blood drawn during study: 60 ml

7.3 Withdrawal of individual subjects

Subjects can leave the study at any time for any reason if they wish to do so without any consequences. The investigator can decide to withdraw a subject from the study for urgent medical reasons.

7.3.1 Specific criteria for withdrawal (if applicable)

7.4 Replacement of individual subjects after withdrawal

Subjects withdrawn during completion of the study will be replaced.

7.5 Follow-up of subjects withdrawn from treatment

All subjects who were withdrawn from the study for medical reasons, will be followed up by the investigator until their care can be transferred to their own physician.

7.6 Premature termination of the study

There are no reasons to terminate this study premature.

8. SAFETY REPORTING

8.1 Section 10 WMO event

In accordance to section 10, subsection 1, of the WMO, the investigator will inform the subjects and the reviewing accredited METC if anything occurs, on the basis of which it appears that the disadvantages of participation may be significantly greater than was foreseen in the research proposal. The study will be suspended pending further review by the accredited METC, except insofar as suspension would jeopardise the subjects' health. The investigator will take care that all subjects are kept informed.

8.2 AEs, SAEs and SUSARs

8.2.1 Adverse events (AEs)

Adverse events are defined as any undesirable experience occurring to a subject during the study, whether or not considered related to metformin. All adverse events reported spontaneously by the subject or observed by the investigator or his staff will be recorded.

8.2.2 Serious adverse events (SAEs)

A serious adverse event is any untoward medical occurrence or effect that at any dose:

- results in death;
- is life threatening (at the time of the event);
- requires hospitalisation or prolongation of existing inpatients' hospitalisation;
- results in persistent or significant disability or incapacity;
- is a congenital anomaly or birth defect;
- Any other important medical event that may not result in death, be life threatening, or require hospitalization, may be considered a serious adverse experience when, based upon appropriate medical judgement, the event may jeopardize the subject or may require an intervention to prevent one of the outcomes listed above.

The sponsor will report the SAEs through the web portal *ToetsingOnline* to the accredited METC that approved the protocol, within 15 days after the sponsor has first knowledge of the serious adverse events.

SAEs that result in death or are life threatening should be reported expedited. The expedited reporting will occur not later than 7 days after the responsible investigator

has first knowledge of the adverse event. This is for a preliminary report with another 8 days for completion of the report.

8.2.3 Suspected unexpected serious adverse reactions (SUSARs)

Adverse reactions are all untoward and unintended responses to an investigational product related to any dose administered.

Unexpected adverse reactions are SUSARs if the following three conditions are met:

1. the event must be serious (see chapter 9.2.2);
2. there must be a certain degree of probability that the event is a harmful and an undesirable reaction to the medicinal product under investigation, regardless of the administered dose;
3. the adverse reaction must be unexpected, that is to say, the nature and severity of the adverse reaction are not in agreement with the product information as recorded in:
   - Summary of Product Characteristics (SPC) for an authorised medicinal product;
   - Investigator’s Brochure for an unauthorised medicinal product.

The sponsor will report expedited the following SUSARs through the web portal *ToetsingOnline* to the METC:

- SUSARs that have arisen in the clinical trial that was assessed by the METC;
- SUSARs that have arisen in other clinical trials of the same sponsor and with the same medicinal product, and that could have consequences for the safety of the subjects involved in the clinical trial that was assessed by the METC.

The remaining SUSARs are recorded in an overview list (line-listing) that will be submitted once every half year to the METC. This line-listing provides an overview of all SUSARs from the study medicine, accompanied by a brief report highlighting the main points of concern.
The expedited reporting of SUSARs through the web portal ToetsingOnline is sufficient as notification to the competent authority.

The sponsor will report expedited all SUSARs to the competent authorities in other Member States, according to the requirements of the Member States.

The expedited reporting will occur not later than 15 days after the sponsor has first knowledge of the adverse reactions. For fatal or life threatening cases the term will be maximal 7 days for a preliminary report with another 8 days for completion of the report.

8.3 Annual safety report

In addition to the expedited reporting of SUSARs, the sponsor will submit, once a year throughout the clinical trial, a safety report to the accredited METC, competent authority, and competent authorities of the concerned Member States.
This safety report consists of:

- a list of all suspected (unexpected or expected) serious adverse reactions, along with an aggregated summary table of all reported serious adverse reactions, ordered by organ system, per study;
- a report concerning the safety of the subjects, consisting of a complete safety analysis and an evaluation of the balance between the efficacy and the harmfulness of the medicine under investigation.

8.4 Follow-up of adverse events

All AEs will be followed until they have abated, or until a stable situation has been reached. Depending on the event, follow up may require additional tests or medical procedures as indicated, and/or referral to the general physician or a medical specialist. SAEs need to be reported till end of study within the Netherlands, as defined in the protocol

9. STATISTICAL ANALYSIS

The baseline characteristics of all subjects will be outlined in a table describing variables such as age, BMI and HOMA-index. The differences in these characteristics before and after the use of metformin will be determined using the mann whitney u test.

9.1 Primary study parameter(s)

P-values for the difference in ^{18}F-FDG uptake in the colon before and after using metformin will be determined using the mann whitney u test.
Differences between lean and obese subjects will also be determined using the mann whitney u test.

9.2 Secondary study parameter(s)

P-values for the difference in core body temperature, energy expenditure, HOMA-index and lactate concentration before and after using metformin will be determined using the mann whitney u test.
Differences between lean and obese subjects will also be determined using the mann whitney u test.

9.3 Other study parameters

Correlations between ^{18}F-FDG uptake and 1) core body temperature, 2) energy expenditure, 3) HOMA-index and 4) lactate concentration will be determined using Spearmans correlation coefficient.

9.4 Interim analysis (if applicable)

None

10. ETHICAL CONSIDERATIONS

10.1 Regulation statement

The study will be conducted according to the principles of the Declaration of Helsinki (Fortaleza, Brazil, October 2013) and in accordance with the Medical Research Involving Human Subjects Act (WMO) and CCMO guidlines

10.2 Recruitment and consent

The healthy volunteers will be recruited by advertisement in a local paper and at the medical faculty of the AMC. When the volunteers contact the study team they will receive additional information about the study and the informed consent procedure will be performed at the screening visit by the research physician L. Bähler. The patient information letter and informed consent form are attached as a separate document. Subjects have access to an independent physician to discuss their participation. They are informed about this opportunity in the patient information letter.

10.3 Objection by minors or incapacitated subjects (if applicable)

No minors or incapacitated subjects will be included in this study

10.4 Benefits and risks assessment, group relatedness

Both obesity and type 2 diabetes are growing worldwide problems. Complications associated with obesity not only cause a high mortality, but also increase health costs[2]. At present, interventions such as diets, sports training, behavioural therapy and pharmacological therapy achieve disappointing and often temporary results.[3-5] The potential of the colon to function as energy dissipating organ could be a new target in the treatment of obesity. The thermogenic properties of the gut have never been explored and it is a new and exciting concept which warrants investigation.

10.5 Compensation for injury

The sponsor/investigator has a liability insurance which is in accordance with article 7, subsection 9 of the WMO.

The sponsor (also) has an insurance which is in accordance with the legal requirements in the Netherlands (Article 7 WMO and the Measure regarding Compulsory Insurance for Clinical Research in Humans of 23th June 2003). This insurance provides cover for damage to research subjects through injury or death caused by the study.

1. € 450.000,-- (i.e. four hundred and fifty thousand Euro) for death or injury for each subject who participates in the Research;
2. € 3.500.000,-- (i.e. three million five hundred thousand Euro) for death or injury for all subjects who participate in the Research;
3. € 5.000.000,-- (i.e. five million Euro) for the total damage incurred by the organisation for all damage disclosed by scientific research for the Sponsor as ‘verrichter’ in the meaning of said Act in each year of insurance coverage.

The insurance applies to the damage that becomes apparent during the study or within 4 years after the end of the study.

10.6 Incentives (if applicable)

All subjects included in this study will receive 230 euro for participating in this study and will receive 0,12 euro/kilometre travelling expenses.

ADMINISTRATIVE ASPECTS, MONITORING AND PUBLICATION

10.7 Handling and storage of data and documents

Each subject will receive a specific subject identification number. The subject will be identified by this number for the duration of the trial. All the participating investigators have access to the source data. Blood samples for analysis and storage will be labelled with this code and visit number. Data will be kept for at least 20 years.

10.8 Amendments

<*The following text is applicable for studies without an investigational medicinal product*.>
Amendments are changes made to the research after a favourable opinion by the accredited METC has been given. All amendments will be notified to the METC that gave a favourable opinion.
All substantial amendments will be notified to the METC and to the competent authority. Non-substantial amendments will not be notified to the accredited METC and the competent authority, but will be recorded and filed by the sponsor.

10.9 Annual progress report

The sponsor/investigator will submit a summary of the progress of the trial to the accredited METC once a year. Information will be provided on the date of inclusion of the first subject, numbers of subjects included and numbers of subjects that have completed the trial, serious adverse events/ serious adverse reactions, other problems, and amendments.

10.10 End of study report

The sponsor will notify the accredited METC of the end of the study within a period of 90 days. The end of the study is defined as the last patient's last visit.

In case the study is ended prematurely, the sponsor will notify the accredited METC within 15 days, including the reasons for the premature termination.

Within one year after the end of the study, the investigator/sponsor will submit a final study report with the results of the study, including any publications/abstracts of the study, to the accredited METC.

10.11 Public disclosure and publication policy

This is an investigator driven study. Data will be published without restrictions by any sponsor

11. STRUCTURED RISK ANALYSIS

11.1 Potential issues of concern

a. Level of knowledge about mechanism of action
See SPC page 6 and 7

b. Previous exposure of human beings with the test product(s) and/or products with a similar biological mechanism
Metformin is used for many years, the use of metformin has been considered as very safe. See SPC page 5 and 6.

c. Can the primary or secondary mechanism be induced in animals and/or in *ex-vivo* human cell material?
The use of metformin is considered as very safe. Furthermore, animal data is not always easy to translate to human data. Since 88% of the subjects using metformin show a high ^{18}F-FDG-uptake in the colon in our retrospective study, we only need a small number of participants to analyze the potential mechanism.

d. Selectivity of the mechanism to target tissue in animals and/or human beings
See SPC page 6

e. Analysis of potential effect
See SPC page 5 and 6

f. Pharmacokinetic considerations
See SPC page 7, 8 and 9

g. Study population
Healthy male subjects aged > 40 years either lean (BMI < 24 kg/m2) or obese (BMI > 28 kg/m2)

h. Interaction with other products
See SPC page 4 and 5

i. Predictability of effect
In our retrospective study, 88% of the subjects using metformin had a high ^{18}F-FDG-uptake in the colon.

j. Can effects be managed?
The change of a lactate acidosis is very small since subjects should be healthy (e.g. no lactate acidosis in medical history, no medication use) and kidney function will be checked. However, when subjects show symptoms of lactate acidosis (acidotic dyspnoe,

abdominal pain and hypothermia) subjects should call the study doctor and contact their general practitioner.

11.2 Synthesis

Metformin is a widely used drug with an established safety profile. The most-occurring side-effect is gastrointestinal discomfort (diarrhoea, bloating, dyspepsia). This is mostly seen at high doses and often ameliorates when using metformin for a longer period. A rare but important complication is lactic acidosis. However, this is most often seen in patients with either renal insufficiency or other underlying causes of impaired lactate metabolism. Therefore, we only include healthy subjects and kidney function will be checked before subjects start with the metformin.
Furthermore, we will use ^{18}F-FDG PET-CT scans to evaluate the glucose uptake in the intestine. The resulting dose from the two radioactive tracers + scans is 7.8 mSv.
The maximal dose for a participant during one year is 10 mSv. Nonetheless, we will strongly emphasize that the participants will not participate in research involving radiation in the two years following this study. Although the change of developing cancer is minimal after participation in this study, we will only include subjects aged over 40 years to minimalize the potential effect of inducing cancer.
Last, the placing of the intravenous canulla in our study can be a unpleasant experience for the subjects and there is a low risk of flebitis at the intravenous injection sites.

Although the methods of our study are accompanied with a moderate risk for healthy subjects, we find this to be justified with adding knowledge to finding a solution for the major obesity and type 2 diabetes epidemic.

12. REFERENCES

1. Swinburn BA, Sacks G, Hall KD, et al. The global obesity pandemic: shaped by global drivers and local environments. Lancet 2011;378:804-14.
2. Toriihara A, Yoshida K, Umehara I, Shibuya H. Normal variants of bowel FDG uptake in dual-time-point PET/CT imaging. Annals of nuclear medicine 2011;25:173-8.
3. Chen CH, Huang MC, Kao CF, et al. Effects of adjunctive metformin on metabolic traits in nondiabetic clozapine-treated patients with schizophrenia and the effect of metformin discontinuation on body weight: a 24-week, randomized, double-blind, placebo-controlled study. The Journal of clinical psychiatry 2013;74:e424-30.
4. Kendall D, Vail A, Amin R, et al. Metformin in obese children and adolescents: the MOCA trial. The Journal of clinical endocrinology and metabolism 2013;98:322-9.

5. Knowler WC, Barrett-Connor E, Fowler SE, et al. Reduction in the incidence of type 2 diabetes with lifestyle intervention or metformin. The New England journal of medicine 2002;346:393-403.
6. Bybel B, Greenberg ID, Paterson J, Ducharme J, Leslie WD. Increased F-18 FDG intestinal uptake in diabetic patients on metformin: a matched case-control analysis. Clinical nuclear medicine 2011;36:452-6.
7. Oh JR, Song HC, Chong A, et al. Impact of medication discontinuation on increased intestinal FDG accumulation in diabetic patients treated with metformin. AJR American journal of roentgenology 2010;195:1404-10.
8. Gontier E, Fourme E, Wartski M, et al. High and typical 18F-FDG bowel uptake in patients treated with metformin. European journal of nuclear medicine and molecular imaging 2008;35:95-9.
9. Ozulker T, Ozulker F, Mert M, Ozpacaci T. Clearance of the high intestinal (18)F-FDG uptake associated with metformin after stopping the drug. European journal of nuclear medicine and molecular imaging 2010;37:1011-7.
10. Bahler L, Verberne HJ, Admiraal WM, Soeters MR, Hoekstra JB, Holleman F. Loss of sympathetic drive may explain loss of brown adipose tissue activity in elderly but not in obese males. EASD abstract # 21 2014.
11. Wilcock C, Bailey CJ. Sites of metformin-stimulated glucose metabolism. Biochemical pharmacology 1990;39:1831-4.
12. Bailey CJ, Wilcock C, Day C. Effect of metformin on glucose metabolism in the splanchnic bed. British journal of pharmacology 1992;105:1009-13.
13. Owen MR, Doran E, Halestrap AP. Evidence that metformin exerts its anti-diabetic effects through inhibition of complex 1 of the mitochondrial respiratory chain. The Biochemical journal 2000;348 Pt 3:607-14.
14. Byrne C, Lim CL. The ingestible telemetric body core temperature sensor: a review of validity and exercise applications. Br J Sports Med 2007;41:126-33.
15. Bouza C, Lopez-Cuadrado T, Gutierrez-Torres LF, Amate J. Efficacy and safety of metformin for treatment of overweight and obesity in adolescents: an updated systematic review and meta-analysis. Obesity facts 2012;5:753-65.
16. Tuomilehto J, Lindstrom J, Eriksson JG, et al. Prevention of type 2 diabetes mellitus by changes in lifestyle among subjects with impaired glucose tolerance. The New England journal of medicine 2001;344:1343-50.
